# Single-Cell Profiling Explore the Immunologic Mechanisms of Tumor Relapse in Hepatocellular Carcinoma

**DOI:** 10.1055/s-0041-1729545

**Published:** 2021-06-11

**Authors:** Liyi Zhang

**Affiliations:** 1 State Key Laboratory of Oncology, Collaborative Innovation Center for Cancer Medicine, Sun Yat-sen University Cancer Center, Guangzhou, Guangdong, People's Republic of China

Despite the worldwide decrease in liver cancer incidence and mortality over the past decade, it is still the fourth leading cause of cancer-related death. 1 The burden of liver cancer is still serious, especially in China. 2 In 2018, more than half of the liver cancer cases and deaths occurred in China. 3 Hepatocellular carcinoma (HCC) is the most common subtype of liver cancer, accounting for 75% of all liver cancer cases. 4 Surgical resection is the first choice for early-stage HCC, but more than 70% of HCC patients develop recurrent cancer within 5 years after surgery, 5 which is the major cause of death in long-term evaluations. Among them, nearly 70% relapsed cases develop an early recurrence within 2 years of surgery. 5 However, the precise molecular mechanisms of rapid recurrence remain unclear.

Tumor immune microenvironment (TIME) consists of various immune cells; education of tumor cells has become a hot topic in cancer research due to its association with initiation, progression, and recurrence. 6 Therefore, profiling the immune contexture of HCC, which is determined by the composition, density, and functional orientation of tumor-infiltrating immune cells, could provide insights into immune escape mechanisms and helpful to design effective therapeutic strategies for recurrent HCC. Single-cell RNA sequencing (scRNA-seq) is the most powerful tool for tumor immune contexture profiling. 7 With broad implications for both basic and clinical cancer research, scRNA-seq has already impacted our conceptual understanding of cancer progression in several cancer types. Although the immune contexture detected by scRNA-seq in primary HCC has been reported, 8 that in early-relapse HCC is still unknown.

In a study recently published in *Cell* , titled “Single-cell landscape of the ecosystem in early-relapse hepatocellular carcinoma,” Sun et al 9 examined 16,498 single-cell transcriptomes from 12 primary and 6 early-relapse HCC tumor tissues by scRNA-seq to define immune contexture at the single-cell resolution level. They found that early-relapse HCC had a distinctive immune contexture that is different from primary HCC. Compared with primary HCC, the density of regulatory T-cells (Tregs) was decreased and those of dendritic cells (DCs) and infiltrated CD8 ^+^ T-cells were increased in early-relapse HCC. Additionally, CD8 ^+^ T-cells in primary HCC displayed a classical exhausted state, but those in early-relapse HCC overexpressed CD161 and resided in an innate-like phenotype with low clonal expansion and cytotoxic state. These alterations were associated with a worse prognosis in HCC. The authors further explored the potential mechanisms of immune escape in early-relapse HCC. They found that tumor cells in early-relapse HCC could evade the host immune system's detection and destruction through suppressing DC-mediated antigen presentation and recruiting CD161 ^high^ CD8 ^+^ T-cells.

The high incidence of early recurrence is the main reason for the poor clinical outcomes of HCC. Ignoring the differences between recurrent and primary tumors, recurrent HCCs are often treated based on the pathological characteristics and molecular classification of the primary HCCs. Although previous studies have reported that similar genomic alterations occurred in both primary and early-relapse HCCs, 10 whether primary and early-relapse HCCs have differences in immune contexture is still unknown. The present study dissected and compared the TIME of primary and early-relapse HCCs by scRNA-seq. Unlike the findings in primary HCC reported in the previous studies (the primary HCC cells recruit Tregs to suppress the immune response 8 ), this study found that Tregs were excluded and CD161 ^high^ CD8 ^+^ T-cells were recruited to create an immunosuppressive microenvironment in early-relapse HCC. In CD161 ^high^ CD8 ^+^ T-cells from recurrent tumor tissues, the expression levels of proliferation- and cytotoxicity-associated genes were significantly reduced and tissue residence–associated genes were increased as compared with those in CD8 ^+^ T-cells from primary tumor tissues. CD161 is reported to be a surface marker for CD8 ^+^ T-cells with innate-like and memory phenotype. 11 In microbial infection, CD161 ^high^ CD8 ^+^ T-cells contribute to the early control of microbial recall infections in both antigen-dependent and -independent manners. 12 Therefore, the adaptive immune response of these cells is considered to be one of the important components of the immune response to the infectious diseases that have ever suffered. 13 However, regardless of the obvious increase of CD161 ^high^ CD8 ^+^ T-cell infiltration, HCC early recurrence can be achieved, which indicates that these T-cells cannot prevent the spread of HCC. The following observations of this study reveal why CD161 ^high^ CD8 ^+^ T-cells with memory phenotypes cannot prevent HCC recurrence: (1) compared with CD161 ^−^ CD8 ^+^ T-cells, CD161 ^high^ CD8 ^+^ T-cells had a weaker antitumor ability, which was manifested as a decrease in the production of granzyme B and (2) T-cell receptor analysis indicated that CD161 ^high^ CD8 ^+^ T-cells in early-relapse HCC reduced clonal expansion and shared most of T-cell receptor clone types with CD8 ^+^ T-cells in primary HCC. These findings indicated that CD161 ^high^ CD8 ^+^ T-cells had the ability to target the clonal neoantigens in primary HCCs, but they could not be effectively activated in early-relapse HCCs.

DCs are critical antigen presenting cells. They play a key role in the regulation of the balance between CD8 ^+^ T-cell immunity and tolerance to neoantigens. In the present study, enhanced effector functions of CD8 ^+^ T-cells were not associated with the increased DC infiltration in early-relapse HCC and suggested that the antigen presentation functions of DCs were impaired in early-relapse HCCs. As we know, CD28 plays a central role in the regulation of T-cell activation and inhibition. 14 Previous study found that programmed death-ligand 1 (PD-L1) has a stronger affinity for CD80 when compared with CD28. 15 As early-relapse HCC exhibited more PD-L1 ^+^ tumor cells than primary HCC, CD80 on a DC might preferentially bind to PD-L1 on early-relapse HCC cells rather than CD28 on a resting T-cell. Therefore, PD-L1 ^+^ recurrence tumor cells may prevent T-cell activation from killing them via reducing CD80–CD28 interactions. If this speculation is true, it means that tumor immunotherapy plus chemotherapy or targeted therapy may be more effective than immune checkpoint blockade as a single agent for patients with recurrent HCC.

Overall, this study provided a comprehensive single-cell profiling in early-relapse HCC and explored the immunologic mechanisms of tumor relapse for the first time. It highlighted the potential significance of targeting immune contexture of HCC, and provided a solid foundation for the development of novel therapeutic strategies for recurrent HCC.
